# Hydrogen Bonding in Liquid Ammonia

**DOI:** 10.1021/acs.jpclett.2c01608

**Published:** 2022-07-28

**Authors:** Aravind Krishnamoorthy, Ken-ichi Nomura, Nitish Baradwaj, Kohei Shimamura, Ruru Ma, Shogo Fukushima, Fuyuki Shimojo, Rajiv K. Kalia, Aiichiro Nakano, Priya Vashishta

**Affiliations:** † Collaboratory for Advanced Computing and Simulations, Department of Chemical Engineering and Materials Science, Department of Physics & Astronomy, and Department of Computer Science, University of Southern California, Los Angeles, California 90089, United States; ‡ Department of Physics, Kumamoto University, Kumamoto 860-8555, Japan

## Abstract

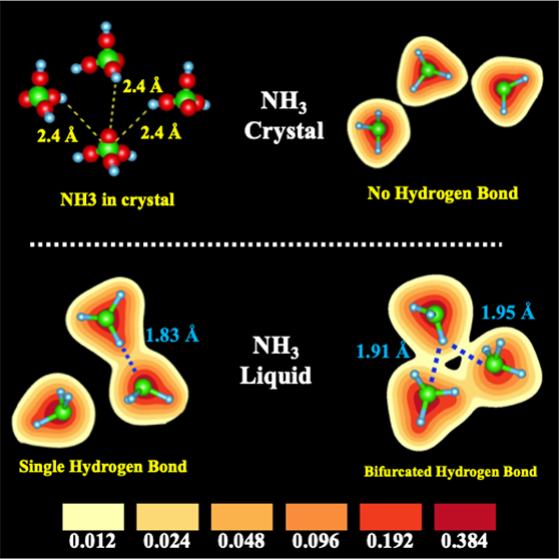

The nature of hydrogen bonding in condensed ammonia phases, liquid and crystalline ammonia has been a topic of much investigation. Here, we use quantum molecular dynamics simulations to investigate hydrogen bond structure and lifetimes in two ammonia phases: liquid ammonia and crystalline ammonia-I. Unlike liquid water, which has two covalently bonded hydrogen and two hydrogen bonds per oxygen atom, each nitrogen atom in liquid ammonia is found to have only one hydrogen bond at 2.24 Å. The computed lifetime of the hydrogen bond is *t* ≅ 0.1 ps. In contrast to crystalline water–ice, we find that hydrogen bonding is practically nonexistent in crystalline ammonia-I.

Ammonia (NH_3_) is intermediate in character between the other two isoelectronic hydrides, water (H_2_O), which forms strongly hydrogen-bonded tetrahedral structures, and methane (CH_4_), that forms close-packed structures at low temperatures. These three materials have four fundamental elements, O, N, C, and H, that are the building blocks of amino acids.^1^ Ammonia (NH_3_) forms a weakly hydrogen-bonded liquid.^2^ It plays a critical role in biochemistry, especially in the structures and functions of proteins.^3^ Water and ammonia are major components of the interiors of the giant icy planets and their satellites. Ammonia is a potentially important source of nitrogen in the solar system and plays a pivotal role in planetary chemistry.^4^ Ammonia in its different forms, green and blue ammonia, is expected to play an important role in production of clean energy and solutions toward climate change.

The concept of hydrogen bonding has played an important role in understanding of the structure of ice and of liquid water as well as other condensed systems.^5^ However, the nature of hydrogen bond and its lifetime in liquid ammonia have remained an enigma.^6,7^ Within the concept of associated liquids, which are characterized by a fluctuating hydrogen bond network, H_2_O is pictured as a three-dimensional distorted tetrahedral network with a 1.8 Å hydrogen bond, while HF is thought to form one-dimensional chain-like structure with the shortest hydrogen bond at 1.6 Å. In contrast, liquid ammonia possesses one of the weakest hydrogen bonds in nature. It is, of course, possible that NH_3_ simply behaves differently in the condensed phase, where environment dependent many-body interactions are important. According to Pimentel and McClellan’s criteria for hydrogen bonding,^8^ a hydrogen bond is said to exist, when (a) there is evidence of a bond and (b) this bond involves a hydrogen atom already covalently bonded to another atom, the condensed-phase evidence for NH_3_ hydrogen bonding is actually much less convincing than that available for HF and H_2_O.

Interest in studying the microscopic structure of liquid NH_3_ is based on the widely held belief that ammonia is, together with HF and H_2_O, one of the simplest H bonded fluids. In fact, the situation is somewhat intriguing because some macroscopic properties of ammonia indicate the presence of a hydrogen bond network in the liquid, while others have a behavior similar to that of simple, nonassociated liquids. For instance, in ammonia there is approximately a 10% increase in relative volume upon melting, whereas it has the opposite sign for H_2_O; ice floats on water! A characteristic property of H bonded fluids is that the range of temperature over which the liquid state exists is larger than in simple fluids. The ratio, *T*_c_/*T*_3_, between the critical temperature, *T*_c_, and the triple point, *T*_3_, is of the order of 2.4 in both HF and H_2_O, while its value is 2.07 for NH_3_, where H bonding is not so well established. Trends in liquid dielectric constant, in entropy of vaporization, and in the effect of methylation on boiling point all distinguish NH_3_ from HF and H_2_O.

The local average structure of pure liquid ammonia has been studied by both X-ray spectroscopy^9^ and X-ray diffraction^10,11^ and neutron diffraction techniques.^12^ Ricci et al. have performed neutron diffraction experiments with isotopic H/D substitution on liquid ammonia at *T* = 213 K and *T* = 273 K, corresponding to densities of 2.53 × 10^–2^ molecules/Å^3^ and 2.26 × 10^–2^ molecules/Å^3^, respectively.^13^ Unlike in H_2_O, where there is a clear hydrogen bonding peak in *g*_OH_(*r*) at ∼1.8 Å, no evidence of a clear peak in *g*_NH_(*r*) for hydrogen bonding was observed in liquid NH_3_. Ricci et al., based on their neutron experiment, concluded the following: “The present study of the microscopic structure of liquid ammonia has shown that the spatial arrangement of nitrogen atoms (NN correlations) indicates that no H-bonded network exists in the liquid at either of the thermodynamic states investigated.”

Theoretical studies of liquid ammonia by molecular dynamics (MD) simulation are numerous,^6,14,15^ and several empirical interaction potential models have been developed.^16^ DFT based quantum MD allows for the investigation systems without empirical interaction potentials.^17^ In this scheme, the forces on the nuclei are computed from an electronic structure “on the fly” within the adiabatic approximation. DFT based MD simulations on crystalline ammonia-I were carried out by Fortes et al.^18^ Diraison et al. investigated liquid ammonia using Car–Parrinello MD^14^ and concluded, “The probability distribution function for a NH_3_ molecule to donate or to accept an HB is very similar. More precisely, for both values of the radial cutoff, about 50% of the molecules are found to accept 1 HB and donate 1 HB, to yield a total of 2 HB per molecule.”

On the basis of DFT based MD simulations, Boese et al.^6^ conclude that “Contrary to earlier conceptions the spatial arrangement of nitrogen atoms showed that no extended hydrogen bonded network exists in liquid ammonia. Nevertheless, some degree of hydrogen bonding was inferred from the temperature dependence of the N–H and H–H radial distribution functions. However, the hydrogen bond interaction in liquid ammonia proved to be much weaker than that in water and no clear hydrogen bond peak was observed in either N–H or H–H correlations, unlike the case of water.”

The question then arises, “Does ammonia hydrogen-bond?”, as was asked in a 1987 *Science* paper by distinguished Harvard theoretical chemist William Klemperer and his collaborators.^19^ They concluded, “If NH_3_ is to be classified as a hydrogen-bond donor, it must be considered a very poor donor, forming weaker, longer, and less linear hydrogen bonds than even HCCH, CF_3_H, and H_2_S.”

Given the discrepancies in the molecular-level understanding of the structure and complexity of the hydrogen bond network in liquid NH_3_, it is important to investigate the nature and structure of the hydrogen bond in liquid ammonia and to determine its lifetime.

Figure 1 shows our computed *g*(*r*) for N–N, N–H, and H–H correlations compared with neutron diffraction results,^19^ demonstrating good agreement with peak positions and widths. Before proceeding further, we emphasize an important point regarding *g*_N–H_(*r*). There are two clear peaks in *g*_O–H_(*r*) in water, the first at 0.95 Å with a coordination of 2 reflecting covalently bonded H atoms in H_2_O, and a second peak at 1.75 Å, also with a coordination of 2, reflecting hydrogen bond in liquid water.^20,21^ In liquid ammonia, there is no such clear peak for hydrogen bonded N···H in *g*_N–H_(*r*). Our goal is to address this unresolved matter and establish the nature of hydrogen bonding in solid and liquid ammonia. Following Pimentel and McClellan’s criteria, we determine the existence of a hydrogen bond between a H and N atom by considering the electron charge density overlap.^22^

**Figure 1:**
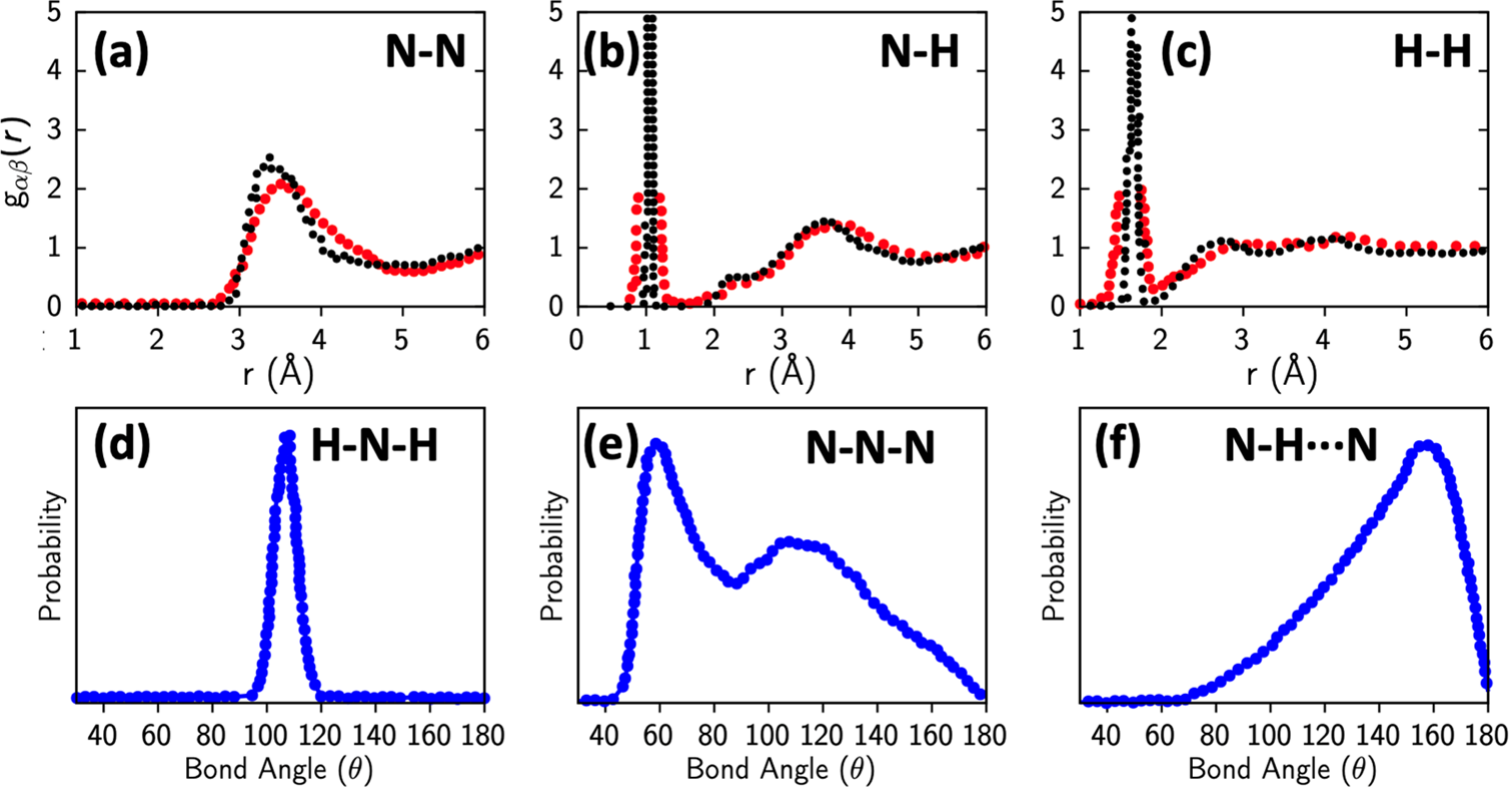
Comparison of structural correlations in liquid ammonia from QMD with neutron scattering experiment: (a–c) Comparison of pair correlation functions, *g*(*r*), of liquid NH_3_ at 213 K using QMD (black) and neutron experiments (red, Boese et al., 2003,^6^ and Ricci et al., 1995^13^). In the *g*_N–H_(*r*), a tiny shallow peak around ∼2.2 Å indicates hydrogen-bonded N and H atoms. The peak heights and their positions for N–H, H–H, and N–N correlations agree well between QMD using the SCAN exchange correlation functional and neutron experiments.^19^ Bond angle distributions from QMD: (d) intramolecular H–N–H covalent bond angle, (e) intermolecular N–N–N bond angle, (f) N–H···N bond angle between intramolecular covalent N–H bond, and the intermolecular H···N hydrogen bond.

We examine the nature of H-bond in crystalline NH_3_ on the basis of charge density overlap rather than simple criterion of bond distance and coordination numbers. It is believed that weak hydrogen bonding between neighboring ammonia molecules results in a pseudo-close-packed arrangement in the crystalline phase. The cubic unit cell of ammonia-I contains four orientationally ordered ammonia molecules on symmetry sites *C*_3*v*_. The dipole moments of the ammonia molecules are directed toward the crystallographic [111] directions. From the crystalline geometry it appears that each molecule both accepts and donates three hydrogen bonds, each of which deviates significantly from the almost perfectly linear hydrogen bonds seen in water–ice.

The crystal structure of ammonia has been interpreted as hydrogen bonded, yet the N–H···N bond angle is not 180° but only 159.3°.^19^ This is a serious problem, since with three hydrogen atoms on each subunit it is difficult to conceive of any reasonable crystal structure without some hydrogen atoms pointed in the general direction of a nitrogen atom. Furthermore, the distribution of angles has a full width at half-maximum of nearly 40°. Thus, it is not obvious that the crystal structure indicates that NH_3_ is an effective hydrogen-bond donor. However, the traditional view has been that the condensed phase interactions of NH_3_ are dominated by hydrogen bonding.

To understand H-bonds in NH_3_, it is important to first understand the structure and coordination around NH_3_ molecules in the crystalline and liquid phases. Figure 2 shows the partial pair correlations for N–H pairs in crystalline NH_3_ (Figure 2a) and in liquid (Figure 2b) along with the coordination around N atoms. There are two important distances in the crystalline NH_3_*g*(*r*) that affects the local structure of NH_3_ molecules. The first peak in *g*_N–H_(*r*) corresponds to the covalent N–H bond at 1 Å which gives a coordination number of 3. The second peak at 2.4 Å corresponds to the distance between N and the nearest H atoms belonging to neighboring NH_3_ molecules. The coordination jumps from 3 to 6 at this distance indicating that three other NH_3_ molecules are equidistant from the central N atom in the first coordination shell at 2.4 Å as shown in Figure 2f. In the liquid phase, there is no significant change in the covalent bonding and the coordination and local structure seen in *g*_N–H_(*r*) up to 1.5 Å are largely intact. However, upon melting, crystalline NH_3_ undergoes a 10% volume expansion, which results in a reorganization of the second shell (coordination of 3 in the crystal) beyond the covalently bonded hydrogen, resulting in a disordered structure as seen in Figure 2b. It is this reorganization of the second shell, which introduces structure even below 2.4 Å, that makes it difficult to classify the nature of the H-bond in NH_3_. This is in contrast to the case of other liquids like H_2_O, where, due to the anomalous but small volume contraction upon melting, the structure of the hydrogen bond is preserved, and the same coordination and symmetry are maintained. To investigate how the three intermolecular hydrogens belonging to the second peak in the crystalline *g*_N–H_(*r*) are reorganized in this disordered peak in Figure 2b, we have plotted their pair distributions separately in Figure 2c–e. It is easy to notice that the nearest intermolecular hydrogen atom, H1 approaches closer than 2.4 Å, while the farthest of the three intermolecular H atoms go beyond 2.8 Å. The second nearest H atom, H2, remains approximately at 2.4 Å, the same distance as in the crystal. To identify if these molecular configurations and intermolecular distances correspond to the existence of hydrogen bonds, we compute and plot electron charge density isosurfaces for crystalline and liquid configurations. The computed charge density in crystalline NH_3_ in Figure 2g shows that the charge density overlap in the intermolecular region is less than 0.012 electron Å^–3^, which is 1/32 the value of 0.384 electron Å^–3^, the charge density value at the center of the N–H covalent bond. We define this value of charge density, which corresponds to binding energies 1000 times weaker than that of a covalent bond, as the threshold for the existence of a H-bond. Using this definition, we notice that H-bonding in crystalline NH_3_ is practically nonexistent. In the liquid phase, the second shell reorganization brings the nearest intermolecular hydrogen closer to the N atom at distances up to 1.8 Å, while simultaneously moving the second- and third-nearest intermolecular hydrogen atoms further away. These liquid configurations demonstrate a strong (>0.012 electron Å^–3^) charge density overlap between the nearest neighbor N–H pair and negligible overlap between the second- and third-nearest neighbor N–H pairs. Therefore, the vast majority of liquid ammonia configurations contain only one hydrogen bond for each NH_3_ molecule. Figure 1d shows that due to the reorganization of the second shell, the second-nearest N–H distances are on average approximately similar to that in the crystal; however, the finite spread in distribution leads to the presence of some second-nearest N–H pairs at distances as low as 1.9 Å, which is comparable to that of first-nearest N–H distances in these configurations. Figure 2j and Figure 2k show computed charge density isosurfaces for configurations with comparable first- and second-nearest intermolecular N–H distances. In both these configurations, the charge overlap in the intermolecular region between both pairs exceeds the threshold of 0.012 electrons Å^–3^ and reveals the existence of transient bifurcated hydrogen bonds, implying that a central NH_3_ molecule is simultaneously H-bonded to its two nearest neighboring NH_3_ molecules.

**Figure 2:**
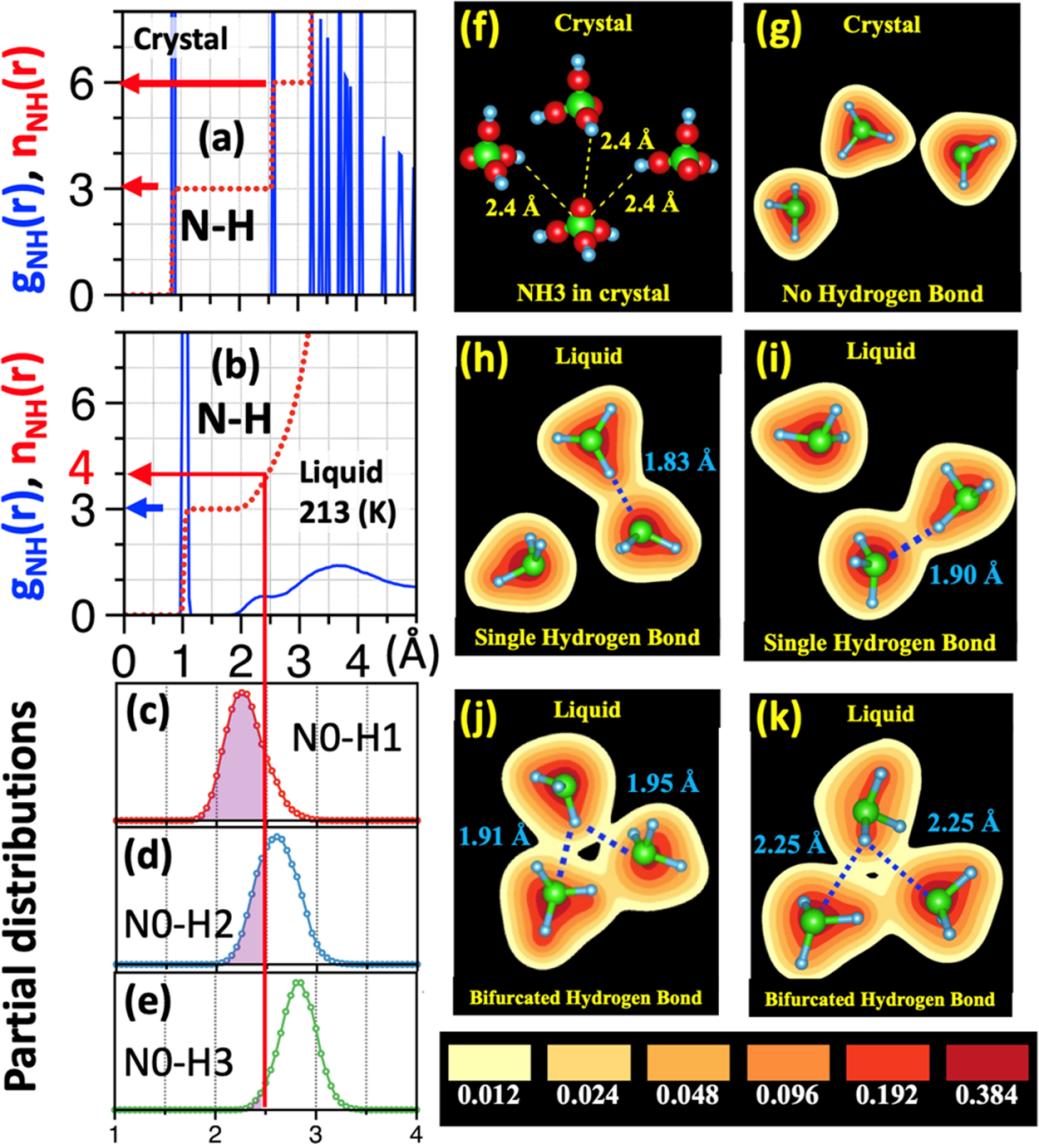
Local coordination and hydrogen bond configurations in crystalline and liquid NH_3_. (a, b) Pair correlation function and coordination numbers for N–H: (a) NH_3_ crystal and (b) liquid NH_3_. (c–e) Partial pair distributions for the N–H pairs, separated into three nearest H atoms, belonging to different ammonia molecules: (c) the nearest H atom, H1, (d) the second-nearest neighbor H2; and (e) third-nearest neighbor, H3. (f) Environment of a NH_3_ molecule in crystal. Crystalline NH_3_ has three equidistant intermolecular H atoms at a distance of 2.4 Å. Charge density overlap in the intermolecular region as computed by SCAN-DFT, (g) charge density is less than 0.012 electrons/Å^3^, which is 1/32 of the charge density overlap of 0.38 electrons/Å^3^, observed in the middle of the covalent N–H bond. Since the energy scales as a quadratic function of charge density, it is ∼1/1000 of the strength of the covalent bond. We define this as the threshold for the existence of a hydrogen bond in NH_3_. The vast majority of molecular configurations (h, i) in QMD trajectories in liquid NH_3_ are characterized by a hydrogen N···H at distances below 1.9 Å. A few configurations (j, k) are found in QMD configurations where there is a single H atom and two N atoms from two other NH_3_ molecules at distances ranging from 1.9 to 2.4 Å. In these cases, the charge density overlap between the H and the neighboring two N exceeds our threshold of 0.012 electrons/Å^3^ and these configurations are considered to have a bifurcated hydrogen bond.

Beyond this unique structure of the H-bond network, several aspects of hydrogen bond dynamics in these systems have also been investigated.^23^ The first inelastic neutron scattering experiments on liquid and solid ammonia were carried out in 1974 by Thaper et al.^24^ Due to limitation of neutron flux and limited resolution, many features in the density of states are not resolved. Another effort to measure the density of states of solid ammonia at 30, 50, 90, and 140 K and liquid ammonia at 210 K was made by Carpenter et al.^12^ at Intense Pulsed Neutron Source at Argonne National Lab. They were able to resolve some features in the density of states; however the background in the data is quite large. Klein and co-workers have used quantum molecular dynamics using the Car–Parrinello scheme to model the structural dynamics of singlet and triplet bipolarons in NH_3_ to identify a novel leapfrog mechanism for bipolaronic diffusion.^21^ Quasi-elastic X-ray scattering experiments have been carried out on liquid ammonia to determine diffusion constants and estimate relaxation time. Inelastic X-ray scattering experiments on high-pressure ammonia liquids in the THz frequency regime revealed that the structural relaxation dynamics of liquid NH_3_ is independent of temperature in the range of 220–298 K, in contrast to what is observed for liquid HF and H_2_O systems, indicating a marked difference in the connectivity of the H-bond network in NH_3_.^10^

We characterize dynamics in liquid ammonia in our QMD simulations by computing H-bond lifetimes using the population time correlation function, *C*^HB^ based on a geometric definition of hydrogen bond for liquid ammonia.
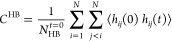
Here, *N* is the number of atoms, *h*_*ij*_(*t*) is unity if two ammonia molecules are hydrogen-bonded at time *t* and otherwise zero, and *N*_HB_^*t* = 0^ is the number of hydrogen bonds at *t* = 0. Figure 3 shows the *C*^HB^ function for QMD at two temperatures, *T* = 213 K and *T* = 233 K. Two ammonia molecules are assumed to be hydrogen bonded if the intermolecular N–H distance is less than 2.4 Å. There is no direct method to experimentally determine the H-bond lifetime.^25^ For example, vibrational relaxation times of 0.74 ps have been reported for water,^26^ whereas observed rotational relaxation times range from 0.6 ps ^27^ to 2.1 ps.^28^

**Figure 3:**
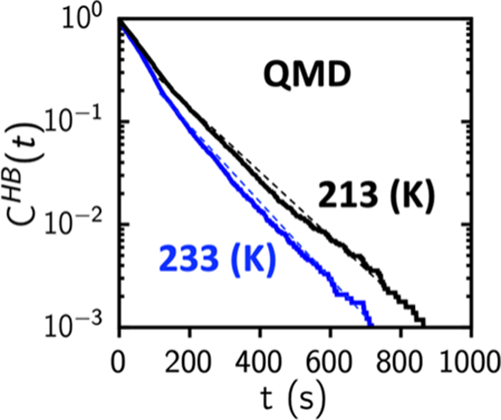
Hydrogen bond lifetimes in liquid ammonia: computed hydrogen bond correlation function for liquid NH_3_*C*^HB^ vs time for QMD trajectories of liquid ammonia. Dashed lines indicate fits of *C*^HB^ to exponential decay to extract H-bond lifetimes at 213 and 233 K from QMD trajectories.

We have examined the rotational relaxation time in liquid ammonia using the characteristic orientational vectors in a NH_3_ molecule. The orientational correlation functions *C*^α^,α ∈ 1, 2, 3, is defined as
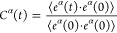
Here *e*^1^ is the unit vector pointing to the direction of molecular dipole moment based on the atomic geometry and empirical charges assigned on each atom position. *e*^2^ is the unit vector pointing from N to H, i.e., the direction of N–H covalent bond, in a NH_3_ molecule. Similarly, *e*^3^ is the one between two H atoms. The relaxation time is obtained by exponential fit, *C*^*α*^(*t*) = exp (−*t*/τ_α_) where τ_α_ is the relaxation time for α-th orientational vector. The top of Table 1 summarized hydrogen bond life times obtained from exponential fits shown in Figure 3, and the bottom half of the Table 1 summarizes the obtained rotational relaxation time. At elevated temperature of 233 K, the three relaxation times are substantially reduced by about a factor of 2.4–2.6, signifying the weak H-bond network in liquid ammonia.

**Table 1:** H-Bond Lifetimes and Orientational Correlations in Liquid NH_3_^a^

H-Bond Lifetime in NH_3_	
*T* (K)	QMD (ps)
213	0.137
233	0.120

Orientational Correlation in NH_3_		
orientational correlation	QMD (ps) (213 K)	QMD (ps) (233 K)
*e*^l^	1.798	0.678
*e*^2^	1.265	0.517
*e*^3^	1.194	0.496

a H-bond lifetimes are computed from C^HB^ decay. H-bond lifetime is estimated to be 0.137 and 0.120 ps in QMD trajectories at 213 and 233 K, respectively. The bottom half of the table summarizes the rotational relaxation time of *e*^*i*^, *i* = *x*, *y*, *z*, the direction of NH_3_ molecular dipole moment along the *i*th direction.

The dynamical correlations in ammonia have been studied through the velocity autocorrelation function, current–current correlation function, and their Fourier transforms. Figure 4a shows the velocity autocorrelation function for deuterated ammonia at 213 K. This is defined as
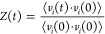
where *v*_*i*_(*t*) denotes the velocity of the *i*th atom at time *t* and the brackets denote the averages over ensembles and atoms. The current–current correlation function for deuterated ammonia is shown in Figure 4b. It is defined as
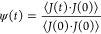
where the charge current is given by *J*(*t*) = ∑_*i*_*Z*_*i*_*ev*_*i*_(*t*). The vibrational density of states is determined by the Fourier transform of the corresponding velocity autocorrelation function.
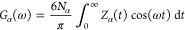
Figure 4c shows the vibrational density of states for deuterated ammonia at 213 K.

**Figure 4:**
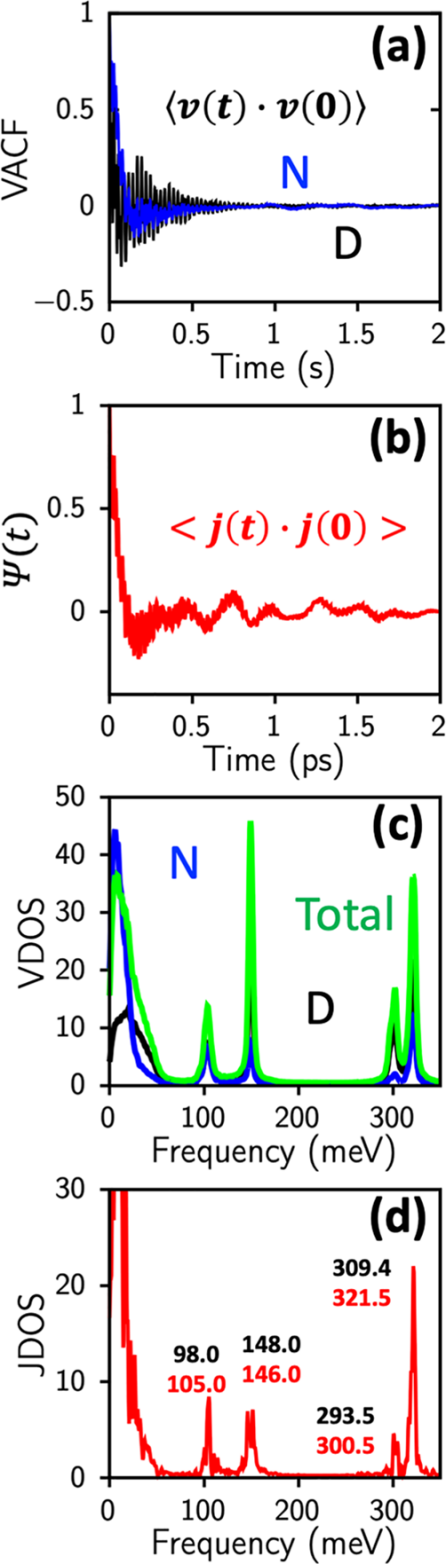
Dynamical correlations in liquid ND_3_ (a) velocity autocorrelation function (VACF) for N and D atoms from QMD of liquid ND_3_ at 213 K. (b) Vibrational density of states from Fourier transform of VACF, with peaks at ∼100 meV, 150 meV, and 300–320 meV. (c) Current–current correlation function for ND3 at 213 K. (d) Fourier transform of current–current correlation function to give the IR spectrum. Experimental peak positions in (d) are indicated in black alongside computed values in red.

The frequency dependent ionic conductivity can be calculated from the Fourier transform of the current–current correlation function
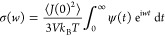
 where *V* is the volume of the system and *k*_B_ is the Boltzmann constant. Figure 4d shows the normalized frequency dependent ionic conductivities for deuterated ammonia at 213 K. Peak positions from IR experimental data are shown in black, and computed values are in red.^29^ Vibrational modes from the total vibrational density of states that obey dipole selection rules are also visible in the compute IR spectrum in Figure 4d.

We have used DFT-SCAN quantum molecular dynamics simulations to investigate the nature of hydrogen bonding in crystalline and liquid ammonia. In contrast to the case of water, with two stable hydrogen bonds per oxygen atom of water molecule, liquid ammonia shows a weaker hydrogen bonding network with only one hydrogen bond per nitrogen atom of each molecule. Hydrogen bonding is found to be practically nonexistent in crystalline ammonia, which, although denser than the liquid phase, has longer intermolecular bonding distances.
